# Physiologically-based pharmacokinetic models versus allometric scaling for prediction of tyrosine-kinase inhibitor exposure from adults to children

**DOI:** 10.1007/s00280-024-04678-0

**Published:** 2024-05-23

**Authors:** Maddalena Centanni, Omar Zaher, David Elhad, Mats O. Karlsson, Lena E. Friberg

**Affiliations:** Department of Pharmacy, Uppsala University, Box 580, Uppsala, 751 23 Sweden

**Keywords:** Pediatric pharmacokinetics, Tyrosine kinase inhibitors, Allometric scaling, Physiologically based pharmacokinetic modeling

## Abstract

**Purpose:**

Model-based methods can predict pediatric exposure and support initial dose selection. The aim of this study was to evaluate the performance of allometric scaling of population pharmacokinetic (popPK) versus physiologically based pharmacokinetic (PBPK) models in predicting the exposure of tyrosine kinase inhibitors (TKIs) for pediatric patients (≥ 2 years), based on adult data. The drugs imatinib, sunitinib and pazopanib were selected as case studies due to their complex PK profiles including high inter-patient variability, active metabolites, time-varying clearances and non-linear absorption.

**Methods:**

Pediatric concentration measurements and adult popPK models were derived from the literature. Adult PBPK models were generated in PK-Sim® using available physicochemical properties, calibrated to adult data when needed. PBPK and popPK models for the pediatric populations were translated from the models for adults and were used to simulate concentration-time profiles that were compared to the observed values.

**Results:**

Ten pediatric datasets were collected from the literature. While both types of models captured the concentration-time profiles of imatinib, its active metabolite, sunitinib and pazopanib, the PBPK models underestimated sunitinib metabolite concentrations. In contrast, allometrically scaled popPK simulations accurately predicted all concentration-time profiles. Trough concentration (C_trough_) predictions from the popPK model fell within a 2-fold range for all compounds, while 3 out of 5 PBPK predictions exceeded this range for the imatinib and sunitinib metabolite concentrations.

**Conclusion:**

Based on the identified case studies it appears that allometric scaling of popPK models is better suited to predict exposure of TKIs in pediatric patients ≥ 2 years. This advantage may be attributed to the stable enzyme expression patterns from 2 years old onwards, which can be easily related to adult levels through allometric scaling. In some instances, both methods performed comparably. Understanding where discrepancies between the model methods arise, can further inform model development and ultimately support pediatric dose selection.

**Supplementary Information:**

The online version contains supplementary material available at 10.1007/s00280-024-04678-0.

## Introduction

Malignancies are one of the major causes of morbidities and mortalities among children in the developed world [1]. Tyrosine kinase inhibitors (TKIs) target specific proteins, receptors and enzymes involved in molecular pathways that are crucial for cancer growth and proliferation and are thus considered safer than traditional chemotherapies [2]. Despite the fact that TKIs are part of the treatment regimen for various solid and hematological malignancies, only a few are approved for pediatric indications [2, 3].

Selection of an adequate dose represents an important step in expanding regulatory approval of TKIs towards the pediatric population. The pediatric regulation by the European Union and the amendment of the Food and Drug Administration’s (FDA) Pediatric Research Equity and Best Pharmaceuticals for Children Act (2006–2007) have instigated more focus on dose optimization in children. During dose selection, differences in both the pharmacokinetics (PK) and pharmacodynamics (PD) of a drug can be taken into account [4]. However, given the redundancy in the mechanism of action of TKIs, differences between children and adults are most likely to originate from discrepancies in drug PK and, as such, exposure matching is desired [5–8]. Indeed, the majority of Phase I investigations in the pediatric population, undertaken for dose selection for subsequent evaluation in Phase II trials concerning safety and efficacy evaluations, have focused on exposure matching with adult patients [9–13], with the exception of crizotinib [14, 15].

Most Phase I studies initially determined the pediatric dose by dividing the fixed adult dosage in milligrams by the average adult body surface area (BSA) of 1.7–1.8 m2, yielding a milligrams per square meter (mg/m²) dose advise [9, 10, 12]. However, not all differences in drug exposure in children compared to adults are due to differences in size, most often expressed as BSA and body weight (WT), but can also be caused by differences in body composition and function, including enzyme ontology [16–18]. It is therefore important to acknowledge these differences when making dose decisions in order to reduce bias in drug exposure. Several model-based methods have emerged over the last years to extrapolate pediatric PK from adults [16, 19, 20], where most focus have been on population PK (popPK) with allometric scaling and physiologically-based pharmacokinetic (PBPK) models [21]. Such models can inform (I) initial dose selection for PK studies and (II) study design in terms of sampling times and sample size based on the expected PK variability.

Population PK (popPK) models serve as mathematical tools to characterize drug exposure and primarily utilize a “top-down” approach by estimating parameters based on observed data from patient populations [19]. Allometric scaling constitutes a technique for the transformation of adult popPK models into pediatric counterparts, achieved through the application of a power exponent. Clearance parameters are typically scaled by a power exponent of 0.75, while a power exponent of 1 is used for volume parameters [22, 23].

PBPK models, on the other hand, adopt a “bottom-up” approach in which plasma and tissue concentration-time profiles can be predicted based on drug-specific characteristics, such as lipophilicity and molecular weight, without the need for human concentration measurements. PBPK models consist of a relatively large number of compartments that represent the different organs of the human body connected by blood flow [20]. These models are well recognized to possess translational utility by mechanistically depicting PK attributes and predicting exposure across diverse patient populations, facilitated by adapting anatomical and physiological aspects of the model to match the pediatric population [20]. For instance, in previous efforts a PBPK model for the TKI nilotinib was used to support the selection of a dose of 230 mg/m^2^ in children between the age of two and eighteen [24]. In earlier studies, allometric scaling has generally been found to perform adequately as compared to PBPK under two scenarios (i) a population older than 2 years for drugs with linear PK and (ii) for individuals younger than 2 years where popPK is possible but a maturation function is necessary [25–27]. The underlying reason being that the activity of enzymes are not fully developed in relation to size until approximately 2 years of age, necessitating the use of maturation functions to account for these developmental differences [25]. Therefore, predictions based solely on allometric scaling can lead to excessively high doses for children below 2 years.

The aim of this study was to evaluate the performance of popPK with allometric scaling and PBPK models in predicting the PK of TKIs for pediatric patients, based on adult models. For this purpose, previously published adult popPK models and the PBPK software PK-Sim were utilized. The drugs sunitinib, imatinib and pazopanib were selected as case studies due to their complex PK profiles, including large variability in exposure with impact on safety and efficacy, presence of active metabolites, time-varying clearance and non-linear absorption [28–31].

## Methods

### Literature search and data collection

A literature search was conducted within the PubMed database using the following search terms: “(sunitinib [Title/Abstract] OR imatinib [Title/Abstract] OR pazopanib [Title/Abstract]) AND (popPK [Title/Abstract] OR (population [Title/Abstract] AND pharmacokinetic [Title/Abstract]))”, (II) “(sunitinib[Title/Abstract] OR imatinib [Title/Abstract] OR pazopanib [Title/Abstract]) AND pharmacokinetic*[Title/Abstract]”, (III) “ (population [Title/Abstract] AND pharmacokinetic [Title/Abstract]) OR ((population [Title/Abstract] AND pharmacokinetic [Title/Abstract]) OR (popPK [Title/Abstract]))” AND pediatric* [Title/Abstract]”, (IV) “PBPK[Title/Abstract] AND pediatric*[Title/Abstract]” on May 2023. Relevant adult popPK publications were selected for potential use.

Adult and pediatric PK data were converted from the available figures into numerical values by the Webplotdigitizer (Version 4.4). Information regarding patient demographics, population size and drug administration protocols were derived from the corresponding publications to recreate the corresponding PK study as accurately as possible. Drug specific physicochemical properties were additionally collected and any missing values were derived from DrugBank, Pubchem, FDA and EMA chemistry reviews.

### popPK models

#### Selection of the adult model

Adult popPK models for each of the three drugs was selected based on several criteria. For imatinib and sunitinib, the model had to describe the PK of both the parent drug and the active metabolite, preferably taking into account the correlations in clearance and volume of distribution. For imatinib, a model describing the increased clearance over time was required, whereas for pazopanib the model had to capture the saturation in drug (dose) absorption. Selected adult popPK models were translated into the R environment (version 4.1.0).

#### Translation of the adult model to the pediatric population

The pediatric PK simulations were conducted by scaling the adult popPK model parameters using allometric principles [23] (Eqs. 1 and 2).

1 $${CL}_{pediatric}={CL}_{adult} x (\frac{{WT}_{i}}{{WT}_{70}}{)}^{0.75}$$

2 $${V}_{pediatric} ={V}_{adult} x (\frac{{WT}_{i}}{{WT}_{70}}{)}^{1}$$

**Fig. 1:**
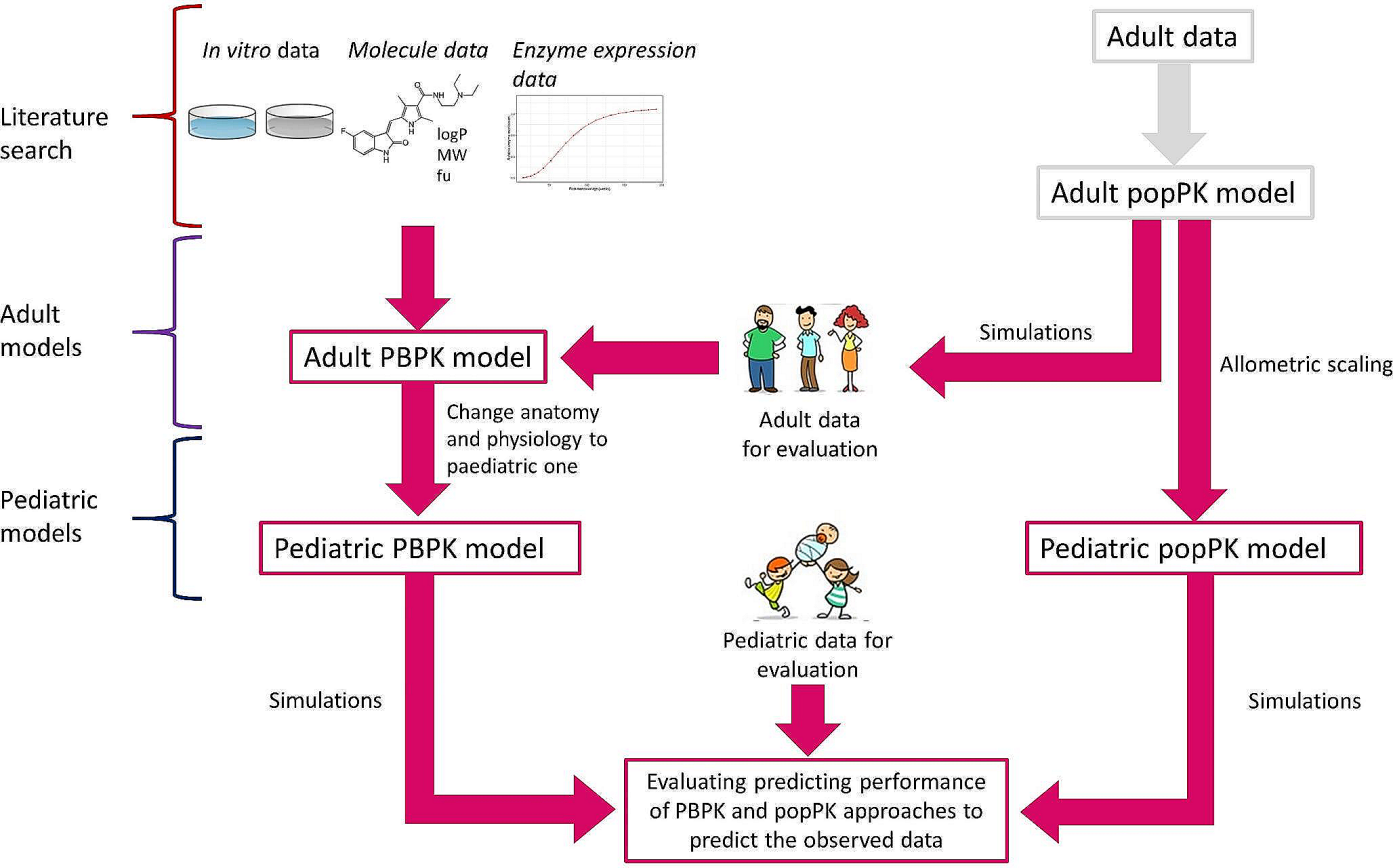
Workflow for developing the pediatric PBPK and popPK models. The pink boxes represent the performed work, remaining components were derived from the literature. *popPK population pharmacokinetic model, PBPK physiologically-based pharmacokinetic model*

Here, the clearance and volume of distribution parameters are normalized by the individual weight of each child (WT_i_) by a reference value (70 kg) and scaled to the population parameters with exponents 0.75 (CL_pediatric_ and Q_pediatric_) and 1 (V_central, pediatric_ and V_peripheral, pediatric_), respectively. The absorption parameter remained unscaled. Simulations were performed using *mrgsolve* (version 0.11.2) in Rstudio (version 4.1.1.). A visual representation of the workflow can be found in Fig. 1.

### PBPK models

#### Building the adult model

An adult PBPK model for each drug case was created within the software PK-Sim (version 9.1). The human body was represented by fifteen organ compartments - each divided into four sub-compartments consisting of plasma, endosomal, interstitial and intracellular space - connected by a systemic circulation [32]. To improve the extrapolation of PBPK models from adult to pediatric populations, the dependence on empirical adult-based PK estimations was minimized by including drug-specific physicochemical properties. Thereby, drug-specific mass-balance information, alongside enzyme maturation data, was integrated. Further details regarding the approach are provided in Sect. 2.3.2 to 2.3.4. If necessary, however, the drug specific properties were optimized to improve the model fit to the adult PK data. Priority was given to build a model that captured the drugs’ trough concentration (C_trough_), as this is used as a biomarker for drug efficacy [2].

The PBPK models were evaluated using data simulated from the adult popPK models. The decision to use simulated data instead of the observed data was prompted by the limitations of the available adult PK datasets, which were derived from a relatively small cohort and only presented as prediction-corrected values. The simulated datasets included 100 virtual individuals, and the patient covariate values were generated by resampling from the initial patient distributions. Sunitinib was administered at a daily dose of 50 mg for fourteen consecutive days. Imatinib was dosed at 400 mg daily for sixty consecutive days. For pazopanib, three different doses (200 mg, 400 mg, and 800 mg per day) were administered for fourteen consecutive days to assess the variations in bioavailability across the doses.

#### Absorption

To capture the process of absorption, information regarding tablet dissolution and the process of drug uptake through the gastro-intestinal tract were incorporated into the model. Firstly, drug dissolution and solubility profiles were obtained from the literature, with priority for values that corresponded to the acidity of the stomach environment (i.e. pH of 1.5 to 3.5). If the predictions were not consistent with the observations, the absorption rate from popPK models was used instead as a starting point. More complex absorption patterns were subsequently evaluated, including a Weibull function and the inclusion of a lag time. Different model descriptions of absorption were evaluated based on the simulated adult data, in conjunction to different partition coefficients and cellular permeability combinations (2.3.3. Distribution section). First-pass metabolism was taken into account via expression of enzymes and corresponding enzyme kinetics within the gastro-intestinal and hepatic tissue (2.3.4. Metabolism section).

#### Distribution

PK-Sim offers five methods to calculate the organ/plasma partition coefficient: Rodgers and Rowland (RR), PK-Sim standard, Poulin and Theil (PT), Schmitt and Berezhkovskiy, and three methods for cellular permeability: PK-Sim standard, charge dependent Schmitt and charge dependent Schmitt normalized to PK-Sim standard. In case none of the combinations gave a satisfactory model output as compared to the observed data, the drug lipophilicity (log(P)) was adjusted based on alternative literature values. Additionally, the model was informed about plasma protein binding, including the corresponding fraction unbound. Sunitinib is an acidic drug and typically binds to albumin [33], whereas imatinib is an alkaline drug that mainly binds to α1-acidglycoprotein [34]. Although pazopanib has alkaline properties it has been found to have a higher affinity for albumin [35].

#### Metabolism and elimination

Drug metabolism was incorporated into the model through the addition of Michaelis-Menten parameter values (i.e. Michaelis constant (K_m_) and maximum rate of reaction (V_max_)) for all relevant enzymes. If no Michaelis-Menten parameter values were available, the reported in vitro CL per mg of liver microsomes was used to enable enzyme-expression related scaling from adults to children.

To integrate enzyme-level drug metabolism with overall metabolism, PBPK models were enhanced by incorporating tissue- and age-specific protein expression data obtained from the Gene Expression Database on the Open Systems Pharmacology website. Three enzyme expression datasets were available: (1) whole genome expression arrays from ArrayExpress (ArrayExpress, 2010), (2) expressed sequence tags (EST) from UniGene and (3) RT-PCR (Reverse transcription polymerase chain reaction) derived gene expression estimates obtained from previous literature [36–38]. The performance of each enzyme expression dataset was evaluated in combination to the different available partition coefficients and cellular permeability calculation methods, yielding a total of 45 possible combinations.

Addition of renal and biliary clearance, both as an empirical clearance value (e.g. CL_renal_) or more mechanistically through the expression of transporters with corresponding Michaelis-Menten parameter, were also assessed.

#### Translation of the adult model to the pediatric population

Once the adult PBPK model was created, the model was translated to a pediatric PBPK model by specifying the underlying age and weight of the population. Enzyme maturation adjustments were integrated using the PK-Sim Ontogeny database. A comprehensive explanation of the enzymes engaged in metabolizing each drug and their maturation patterns with age is available in Online Resource 1. Likewise, changes in anatomical and physiological features are automatically generated by the PK-Sim software. A visual representation of the workflow can be found in Fig. 1.

### Evaluating model performance

The final pediatric popPK and PBPK models were used to simulate PK observations that represent the studies of the observed pediatric data in terms of patient WT and age, drug dosing, sampling times, number of individuals and proportion of males versus females. In case of missing weights and BSA values, the reported World Health Organization (WHO) child growth standards were used for the corresponding age and sex [39]. For each model, the original dataset was re-simulated 200 times and summarized in a visual-predictive check (VPC) with the observed data [40].

## Results

### Literature search and data collection

A total of ten pediatric PK studies were identified (Table 1). Nine datasets were available for imatinib and four datasets for sunitinib. For pazopanib, only data from the tablet formulation were included, whereas the plasma concentration measurements from the suspension formulation were omitted. Unfortunately, the pazopanib measurements were not reported as observations, but only as mean and standard deviation for each distinct time points and dosage level. Therefore, to generate observations, ten data points were randomly sampled for each reported time point for each individual using the provided distribution.

**Table 1:** Summary of pediatric PK studies

Drug	Number patients	Dose	Age range	Sample	Sample times	Weight (kg)	BSA (m2)	Sex (% Male)	Reference
**Imatinib**									
	4	300 mg bid	2–18	C_trough_	Day 1: 12. Day 8: 0 (hours)	*	*	75	Baruchel et al. [34]
	1	500 mg	2–18	C_trough_	Day 1: 12. Day 8: 0 (hours)	*	*	100	Baruchel et al. [34]
	26	300 mg/ m^2^	4–17	C_trough_	*Day 8*	*	*	76.9	Jaeger et al. [34]
	4	300 mg/ m^2^	6–15	Full PK	*Day 1 and 8*: 0.5, 1, 2, 4, 8, 24 (hours)	*	*	50	Marangon et al. [54]
	31	260 mg/ m^2^	3–20	Full PK	*Day 8 (cycle 1)*: 0, 0.5, 1, 1.5, 2, 4, 8, 24 (hours)	*	*	74	Champagne et al. [9]
	31	340 mg/ m^2^	3–20	Full PK	*Day 8 (cycle 1)*: 0, 0.5, 1, 1.5, 2, 4, 8, 24 (hours)	*	*	74	Champagne et al. [9]
	6	440 mg/ m^2^	6–22	Full PK	Day 1: 0.5, 2, 1–3, 6–9, 12 (hours)	50 (14.6–119.2)	*	65.4	Menon-Andersen et al. [46]
	41	440 mg/ m^2^	6–22	C_trough_	Day 1, 8, 18	50 (14.6–119.2)	*	65.4	Menon-Andersen et al. [46]
	33	340 mg/ m^2^	2–22	Full PK	*Day 1*: 1, 3, 5, 7, 12, 24 (hours). *Day 30 and 60*: 0, 2, 4 (hours)	38 (12–80)	*	60.7	Petain et al. [55]
**Pazopanib**	6	275 mg/m2	5.0-21.7	C_trough_	*Cycle 1*: 1, 13–17, 20–24, 26–28. Odd-numbered cycles: 0 (days)	*	*	52	Glade Bender et al. [56]
	6	350 mg/m2	5.0-21.7	C_trough_	*Cycle 1*: 1, 13–17, 20–24, 26–28. Odd-numbered cycles: 0 (days)	*	*	52	Glade Bender et al. [56]
	5	450 mg/m2	5.0-21.7	C_trough_	*Cycle 1*: 1, 13–17, 20–24, 26–28. Odd-numbered cycles: 0 (days)	*	*	52	Glade Bender et al. [56]
	5	600 mg/m2	5.0-21.7	C_trough_	*Cycle 1*: 1, 13–17, 20–24, 26–28. Odd-numbered cycles: 0 (days)	*	*	52	Glade Bender et al. [56]
	6	275 mg/m2	5.0-21.7	Full PK	*Day 1, 15–17, 22–24*: 0.5, 1, 2, 4, 6, 8, 10 to 12, and 24–26 (hours)	*	*	52	Glade Bender et al. [56]
	6	350 mg/m2	5.0-21.7	Full PK	*Day 1, 15–17, 22–24*: 0.5, 1, 2, 4, 6, 8, 10 to 12, and 24–26 (hours)	*	*	52	Glade Bender et al. [56]
	5	450 mg/m2	5.0-21.7	Full PK	*Day 1, 15–17, 22–24*: 0.5, 1, 2, 4, 6, 8, 10 to 12, and 24–26 (hours)	*	*	52	Glade Bender et al. [56]
	5	600 mg/m2	5.0-21.7	Full PK	*Day 1, 15–17, 22–24*: 0.5, 1, 2, 4, 6, 8, 10 to 12, and 24–26 (hours)	*	*	52	Glade Bender et al. [56]
**Sunitinib**	6	15 or 20 mg/m2	2–5	Full PK, C_trough_	*Dose 1*: 1, 2, 4, 6, 6–8, 8–10, 24–28, and 48–52 (hours). *Ctrough cycle 1*: 1, 7, 14, 15, 21, 28 (days). *Ctrough cycle 2/3*: 1, 28 (days)	18.3 (16.2–28.7) range	0.69 (0.66–0.98) range	50	Wang et al. [57, 58]
	20	15 or 20 mg/m2	6–11	Full PK, C_trough_	*Dose 1*: 1, 2, 4, 6, 6–8, 8–10, 24–28, and 48–52 (hours). *Ctrough cycle 1*: 1, 7, 14, 15, 21, 28 (days). *Ctrough cycle 2/3*: 1, 28 (days)	28.4 (17.1–56.3) range	1.10 (0.72–1.48) range	45	Wang et al. [57, 58]
	27	15 or 20 mg/m2	12–17	Full PK, C_trough_	*Dose 1*: 1, 2, 4, 6, 6–8, 8–10, 24–28, and 48–52 (hours). *Ctrough cycle 1*: 1, 7, 14, 15, 21, 28 (days). *Ctrough cycle 2/3*: 1, 28 (days)	60.4 (37.1–100) range	1.63 (1.27–2.14) range	40.7	Wang et al. [57, 58]
	6	15 or 20 mg/m2	18–21	Full PK, C_trough_	*Dose 1*: 1, 2, 4, 6, 6–8, 8–10, 24–28, and 48–52 (hours). *Ctrough cycle 1*: 1, 7, 14, 15, 21, 28 (days). *Ctrough cycle 2/3*: 1, 28 (days)	71.3 (62.5–74.5) range	1.87 (1.62–1.92) range	50	Wang et al. [57, 58]

* value not reported. bid = bidaily

Table 2 provides an overview of the physicochemical properties of each drug, as well as other relevant parameters related to drug absorption, distribution, metabolism, and elimination.

**Table 2:** PBPK input parameter values

Parameter	Units	Sunitinib	SU12662	Imatinib	NDMI	Pazopanib	Reference
MW	(g/mol)	398.48	370.4	493.6	459.7	437.53	[34, 58, 59]
LogP	-	3.1	1.88	4 *d	2 *c	1.9	[34, 58, 60]
Solubility	mg/ml	25 (ph = 6.8)	-	0.05	0.006	0.00433	[61–63]
pKa1	-	8.5	10.96	8.07	12.69	2.1	[58, 59, 62]
pKa2	-	-	-	3.73	9.23	6.4	[59, 62]
pKa3	-	-	-	-	-	10.2	[59]
CYP enzyme database	-	RT-PCR	RT-PCR	EST	EST	EST	*d
Absorption profile	-	Weibull (half-life 200 min, dissolution shape 2)	-	Weibull (half-life 20.74 min, dissolution shape 2)	-	Weibull (half-life 60 min, dissolution shape 1)	*d
Vmax, CYP3A4	nmol/min/nmol	110	110	3	3 *c	-	[34, 64]
Km, CYP3A4	uM	32	32	10.54	10.54 *c	-	[34, 64]
Vmax, CYP3A5	nmol/min/nmol	97	97	-	-	-	[64]
Km, CYP3A5	uM	35	35	-	-	-	[64]
Vmax, CYP2C8	nmol/min/nmol	-	-	56.4	56.4 *c	-	[34]
Km, CYP2C8	uM	-	-	7.49	7.49 *c	-	[34]
CL_int_ (µl.min^− 1^.pmol enzyme-1) CYP3A4		-	-	3.34 × 10 ^−8^	3.34 × 10 ^−8^ *c	-	[34]
CL_int_ (µl.min^− 1^.pmol enzyme^− 1^) CYP2C8		-	-	2.42 × 10 ^−8^	2.42 × 10 ^−8^ *c	-	[34]
Clint (µl.min-1.mg mic. protein-1) CYP3A4	-	-	-	-	-	65 *d	[45]
Cellular Permeability	-	Schmitt normalized to PK-Sim standard	Schmitt normalized to PK-Sim standard	Berezhkosvskiy	Rogers and Rowlands	Berezhkosvskiy	*d
Partition coefficient	-	PK-Sim standard	PK-Sim standard	PK-Sim standard	PK-Sim standard	PK-Sim standard	-
Plasma Binding component	-	Albumin	Albumin	α1-acid-glycoprotein	α1-acid-glycoprotein *c	Albumin	[33–35]
Fraction unbound	-	0.05	0.1	0.05	0.05 *c	0.001	[34, 35, 58]

a) Accessed from pubchem.ncbi.nlm.gov

b) Accessed from Drugbank

c) Assumed to be the same as parent

d) Estimated in PK-Sim

### popPK models

Three popPK models were identified for sunitinib. The model by Yu et al. was selected as it captured the PK of both sunitinib and the active metabolite SU12662, including covariance between their parameters [29].

For imatinib the baseline model by Delbaldo et al. was selected, which described the PK of both imatinib as well as the active metabolite N-desmethyl-Imatinib (NDMI) on day 1 [41]. An increase in clearance in imatinib was added as a linear relative increase of 0.567% per day (giving rise to the reported average increase of 17% at day 30 [42], 34% at day 60 [43]), where time is a continuous variable:

3 $${CL}_{pediatric}={CL}_{adult} x (\frac{{WT}_{i}}{{WT}_{70}}{)}^{0.75} x {(1+0.00567) }^{TIME\left(days\right)}$$

For pazopanib, the model by Yu et al. [30] described the nonlinear absorption of the drug and different doses and was therefore selected. No further adjustments were made to the absorption structure, thus assuming that the dose-related absorption saturation occurred at the same absolute dose in children as in adults:

4 $${F}_{pediatric}={F}_{adult} =1- \frac{{E}_{max} x (Dose\left(mg\right)-200)}{ED50 + (Dose\left(mg\right)-200)}$$

Where E_max_ (with a value of 1) represents the maximum effect of dose on bioavailability and ED_50_ (with a value of 480 mg) represents the dose level minus 200 mg at which the bioavailability is half. Allometric scaling of the clearance and distribution parameters was not part of the popPK models of imatinib and pazopanib and was therefore added for the pediatric simulations using a reference body weight of 70 kg.

### PBPK models

#### Absorption and distribution

Since solubility and dissolution profiles did not give rise to the observed absorption profiles, a Weibull function was fitted for each drug, using the absorption rate constant from the popPK models as a starting point for the absorption half-life (Table 2). For pazopanib, the bioavailability at higher doses were overestimated despite informing the model on low tablet solubility. The evaluated adult (200, 400 and 800 mg) doses were therefore lowered proportionally to the bioavailability in the popPK model (Eq. 4), which resulted in adequate predictions of the observed data (Online Resource 1) [30]. For the pediatric population, the same proportional dose reduction was done using for the evaluated pediatric doses 275, 350, 450 and 600 mg/m^2^, assuming a BSA of 1 m^2^.

For all compounds, the partition coefficient, as calculated by the PK-Sim standard method, gave the best results. For imatinib, the initial logP of 1.99 [31] was increased to 4.0 to improve the model simulations, which is similar to the reported logP of 3.5 [44].

#### Metabolism and elimination

For sunitinib and imatinib, in vitro parameters of CYP metabolism, the Michaelis-Menten parameters were available for the parent compounds, but not for their active metabolites, SU12662 and NDMI. Therefore, the values for the parent compounds were employed for the metabolites. For pazopanib, no enzyme-specific values were available, and therefore, drug metabolism was incorporated through intrinsic clearance (Cl_int_), which was based on drug clearance per total grams of CYP3A4 protein. The pazopanib CL_int_ was optimized based on the observed data to 65 µl/min/mg microsomal protein, which is significantly lower than the reported optimized CL_int_ of 175 µl/min/mg microsomal protein [45].

The PBPK simulations of the final adult models can be found in the Online Resource 1. For imatinib, the model captured the adult PK profile well, but had a tendency to underestimate the NMDI concentration-time profile. For sunitinib, the PBPK model underestimated maximum concentrations (C_max_), but captured C_trough_ of the concentration-time profiles, particularly at steady state. For the active metabolite SU12662, the simulations underestimate the adult PK profiles. In case of pazopanib, the PBPK model captured the median PK profile well, but underestimated the population variability.

### Pediatric simulations

**Fig. 2:**
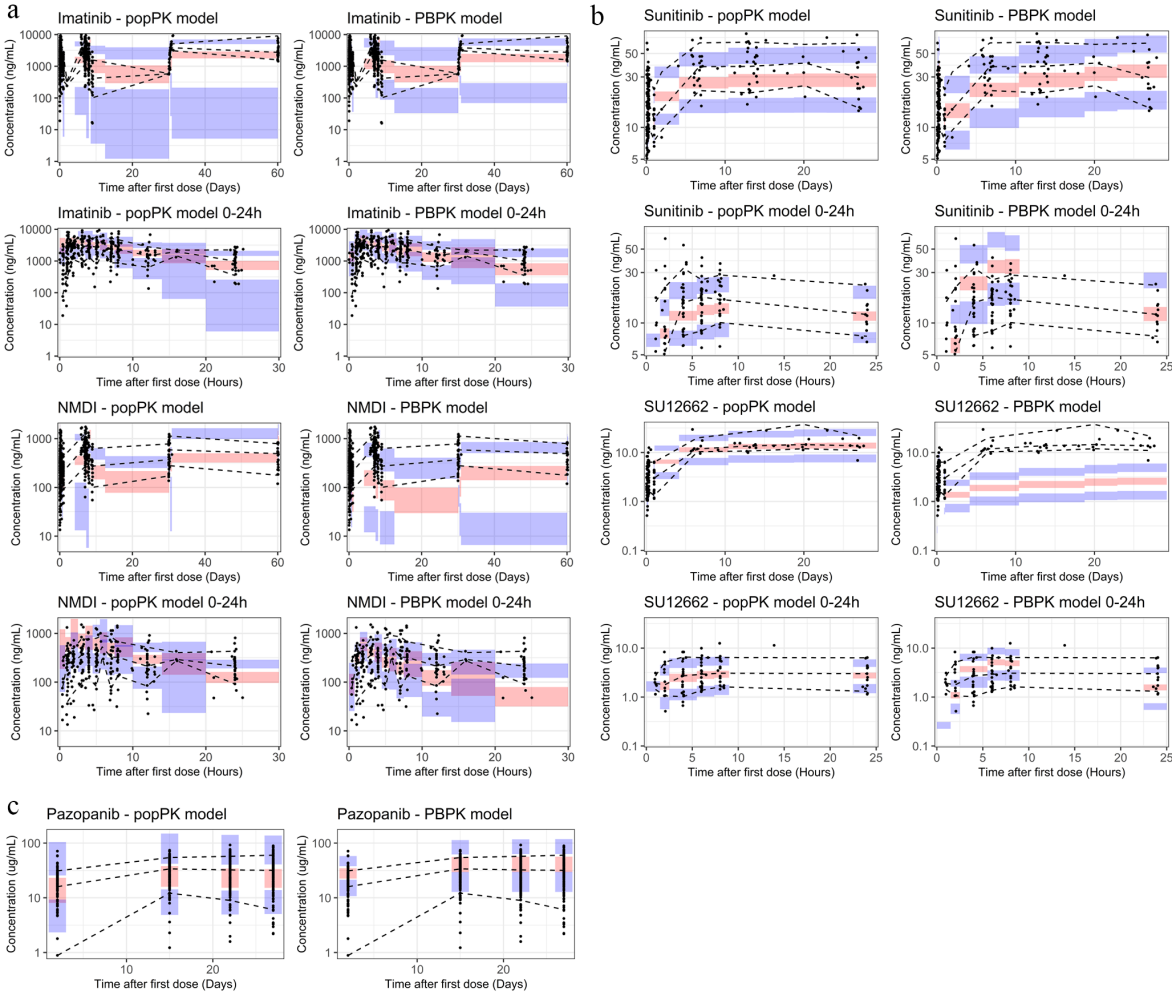
Visual predictive checks for the pediatric PBPK and popPK models. The solid black circles (•) represent observed drug concentrations. The dashed black lines (---) are the median, 10th and 90th percentiles of the observed data. The shaded areas correspond to the 95% confidence intervals for the median (red), 10th (blue) and 90th (blue) percentiles based on the simulated data (*n* = 200)

In the VPCs of imatinib, both computational models exhibited moderate overestimation of the C_max_ and underestimation of the C_trough_ in the median, the 2.5th and the 97.5th percentile in the 24-hour concentration profiles after first dose (Fig. 2a). At steady state, both models generally demonstrated accurate predictions of steady-state median C_trough_ values, but underpredicted the steady-state 2.5th percentile. This is reflected back in the NMDI profiles, where both the PBPK and popPK models moderately overestimated the C_max_ and underestimate the C_trough_ for the median, the 2.5th and the 97.5th percentiles after the first dose (Fig. 2b). The steady-state C_trough_ NMDI values was underpredicted by the PBPK model, whereas the popPK model captures the time-course but underestimates the variability. Both models overestimate the 2.5th percentile for NMDI concentration over time.

Both the PBPK and popPK models simulated concentration-time profiles that underpredicted the observed pediatric data over multiple dosing occasions of sunitinib, but captured the C_trough_ at 28 days (672 h) (Fig. 2c). For the first 24-hour concentration profiles after first dose of sunitinib, the PBPK model tended to overestimate the initial profile, while the popPK model underpredicted the exposure, but both models effectively predicted steady-state C_trough_ concentrations. For SU12662, the PBPK model generated concentrations markedly lower than the observed data, while the popPK model captured both the PK data but overestimated the variability (Fig. 2d). For the first 24-hour concentration profiles after first dose of sunitinib, the popPK model captures the full concentration-time profile of SU12662, whereas the PBPK model tends to overestimate the concentration at C_max_ and underestimate the concentration at C_trough_.

In the case of pazopanib, simulations produced by the popPK and the PBPK model captured the observed data, but overestimated the 2.5th percentiles concentration observed during the first dose administration. Additionally, the PBPK model generally overestimated the concentrations at the 2.5th and 97.5th percentile (Fig. 2e).

The ratio of predicted C_trough_ values after one dose and at steady-state and observed values are presented in Table 3. Using the threshold of 2-fold prediction error in exposure, the PBPK model underestimated the C_trough_ for active metabolites NMDI and SU12662.

**Table 3:** C_trough_ values (ng/ml)

	Observed (C_trough_, ng/ml)	Observed (C_trough, ss_, ng/ml)	PBPK/Observed (C_trough_, ratio)	PBPK/Observed (C_trough, ss_, ratio)	popPK/Observed (C_trough_, ratio)	popPK/Observed (C_trough, ss_, ratio)
Imatinib	847.8	3306.6	1.0	1.0	1.0	1.0
NMDI	186.3	449.6	0.4	0.9	0.7	1.9
Sunitinib	12.7	35.3	0.7	1.1	0.9	0.9
SU12662	3.1	13.4	0.3	0.2	0.9	1.2
Pazopanib	16.6	32.1	1.8	1.5	1.5	1.1

## Discussion

This investigation evaluated the performance of popPK models with allometric scaling and PBPK models in predicting the PK of TKIs for pediatric patients, using three TKIs with intricate PK characteristics and administered to children across diverse age and dosing ranges. Allometric scaling of popPK models predicted exposure within 0.5-2 fold the observed values for all TKIs. On the other hand, PBPK-based predictions underpredicted the exposure of the active metabolites, thus resulting in potentially larger miscalculations of initial dose selection.

For imatinib, both the popPK and PBPK models tended to overestimate the C_max_ of children in the first 0–24 h time profiles, particularly for the active metabolite NMDI, but captured the C_trough_ values adequately (Fig. 2). This could potentially be due to differences in the absorption rate between adults and children that are not well represented by both models, although the reported duration of absorption estimated for children resembled that of adults at 1.67 [46] and 1.5 h [47], respectively. Regarding sunitinib, both models successfully simulated PK profiles that resemble the observed data; however, the PBPK model generated a higher C_max_ in the 24-hour profile (Fig. 2). This phenomenon is, however, already witnessed in the simulations arising from the adult PBPK model compared to the observed data, and therefore regarded as a constraint within the PBPK model rather than an issue of extrapolation. Similarly, the PBPK projections for the active metabolite SU12662 were initially lower than the observed data in adults, indicating that underestimation of the pediatric data is not related to inadequate extrapolation. Regarding pazopanib, the PBPK model demonstrated an overestimation of observed pediatric concentrations in the 2.5th and 95th percentile (Fig. 2), potentially stemming from an overestimated bioavailability at elevated doses. This might be linked to an overestimation of the absorbed fraction from the gastrointestinal tract in children, considering the low first-pass effect for pazopanib [48].

As highlighted in the introduction section, model-based predictions could be employed to (I) inform first dose selection [49, 50]. For the Phase I PK studies of TKIs, a maximum of 2-fold deviation from the adult exposure was deemed acceptable [9–13]. Based on this limit, the C_trough_ PK predictions were considered acceptable for the popPK model whereas in 2 out of 5 cases the PBPK predictions were outside the 2-fold range (Table 3). Therefore, while previous research has demonstrated that both allometric scaling and PBPK models produced predictions within the accepted prediction error for children (≥ 2 years) based on data from adults [21, 25, 26, 49, 51, 52], our findings suggest that extrapolations based on allometric scaling might perform better for compounds with complex PK. However, considering that our evaluation focused on only three compounds out of > 80 TKIs that have received regulatory approval [53], the generalizability of our findings to all available TKIs remains limited. Additionally, it should be noted that a 2-fold prediction error remains large for TKIs, in which exposure is crucial for drug efficacy and safety and thus a lower deviation is desired [2, 28]. Based on the results for pazopanib, the PBPK model overpredicted the concentration by 50%, whereas the popPK model had an overprediction of 10%. Therefore, it is anticipated that PBPK recommendations would suggest lower dose for evaluation in a pediatric PK study as compared to those derived from the popPK model.

An additional purpose of predictions based on models is to (II) inform study design [49, 50]. Given that drugs like imatinib, sunitinib, and pazopanib have demonstrated considerable variability in exposure levels among different patients [28], this variability can impact the necessary sample size for a pediatric PK study focused on achieving comparable exposure levels [5]. Hence, it is imperative that model-based predictions capture the variability within PK profiles, a requirement that both PBPK and popPK models have largely fulfilled in the context of this investigation. Given that the principal objective of forecasting pediatric PK profiles is to facilitate informed dose selection, such computational forecasts can serve as a valuable instrument within this decision-making process [49].

In addition to the comparison between simulated and observed profiles, there exist various pragmatic considerations to account for when selecting PBPK and popPK models. Both the PBPK and popPK methodologies rely upon the availability of adult-centric data, however, the PBPK model necessitating supplementary in vitro data to facilitate model generation. Furthermore, the capacity of PBPK models is constrained by software limitations. Consequently, if the essential features are absent within the PBPK software, it might be more feasible to integrate mechanistic components within a popPK model. In the context of TKIs, it is anticipated that factors such as the type of disease and the extent of tumor burden could influence PK. While the concept of “physiologically-based” underscores the capability to extrapolate adult anatomical and physiological attributes into pediatric models, if pivotal structural elements are deficient within the model or if the pathways of elimination are inadequately established the translational capacity might not exceed that of a popPK approach [21].

There exist several limitations within this study that merit emphasis. Concerning the PBPK models, the incorporation of time-dependent clearance and dose-dependent bioavailability was added empirically due to inherent software constraints, thereby relying on the assumption that there was concordance between pediatric and adult subjects. Notably, for pazopanib, this approach led to an overestimation of the higher concentrations. A PBPK software that accommodates mechanistic integration of dose-dependent bioavailability is anticipated to enhance model predictability. Furthermore, due to the absence of individual-level data, the differentiation between age and dose categories was unfeasible during simulations, thereby precluding subgroup analyses aimed at identifying factors that could have influenced model misrepresentation.

In summary, based on the studied TKI cases, it appears that allometric scaling of popPK models could be more suitable for predicting exposure in pediatric patients aged 2 years and older. This is likely due to the model’s ability to account for TKIs with complex PK characteristics. Given the limited amount of drug cases, however, the generalizability of our findings to all available TKIs remains limited. One potential strategy to address the existing uncertainty in model-guided TKI exposure projections is to use both methods simultaneously, to evaluate if discrepancies arise in projections for specific compounds. In such instances, an additional investigation could be performed to identify the origins of discrepancies in order to understand how they lead to distinct projections between the two approaches.

## Electronic supplementary material

Below is the link to the electronic supplementary material.

Supplementary Material 1

## Data Availability

No datasets were generated or analysed during the current study.

## Code availability

The code used for data analysis and statistical modeling is available upon request from the corresponding author L.E.F..
