# Supplementary material for: Physiologically-based pharmacokinetic models versus allometric scaling for prediction of tyrosine-kinase inhibitor exposure from adults to children
Source: Cancer Chemother Pharmacol. 2024 May 23;94(2):297–310. doi: 10.1007/s00280-024-04678-0 (PMC11390758; doi:10.1007/s00280-024-04678-0)
Supplement: Supplementary file 1 — Supplementary Material 1 [file 280_2024_4678_MOESM1_ESM.docx]

**Physiologically-based pharmacokinetic models versus allometric scaling for prediction of tyrosine-kinase inhibitor exposure from adults to children**

Maddalena Centanni^1^, Omar Zaher^1^, David Elhad^1^, Mats O. Karlsson^1^, Lena E. Friberg^1^*

**Affiliations**

1. Department of Pharmacy, Uppsala University, Uppsala, Sweden.

*Corresponding author:

Lena E. Friberg

Dept of Pharmacy

Uppsala University, Box 580

751 23 Uppsala

Sweden

lena.friberg@farmaci.uu.se

ORCID ID: 0000-0002-2979-679X

**Enzyme maturation**

**CYP3A4 expression with age in PK-Sim* (imatinib, sunitinib and pazopanib metabolism)**


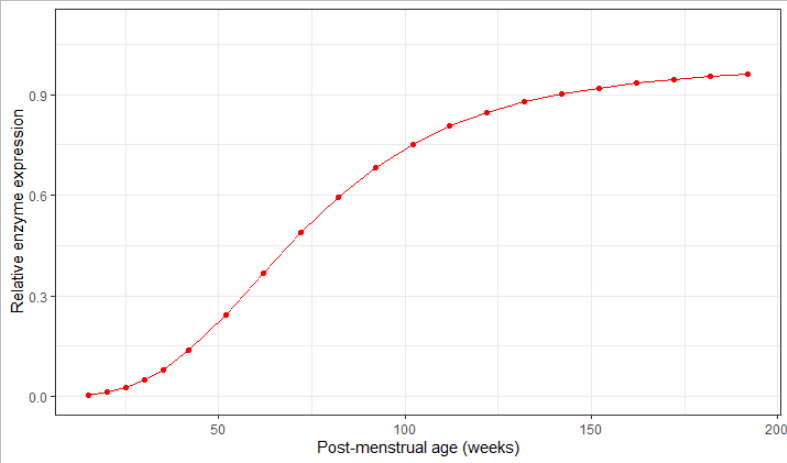

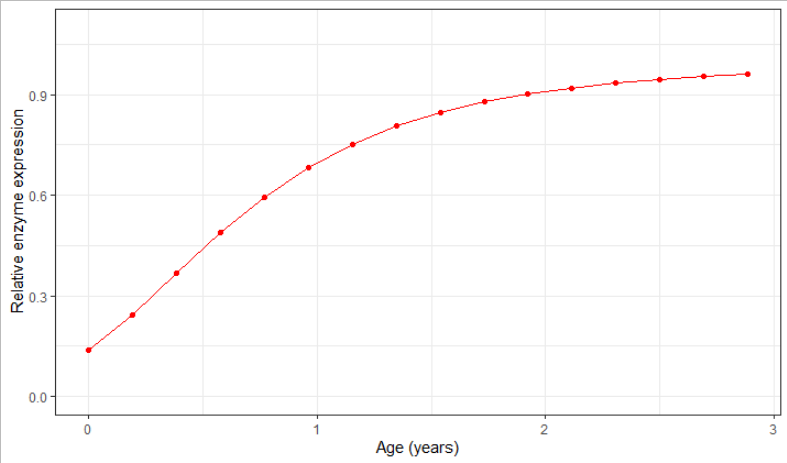


**CYP3A5 expression with age in PK-Sim* (sunitinib metabolism)**

Given the data's variability, it was not feasible to establish an ontogeny fit for CYP3A5 (1). Consequently, the ontogeny function for this enzyme remains constant at 1 across all age groups.
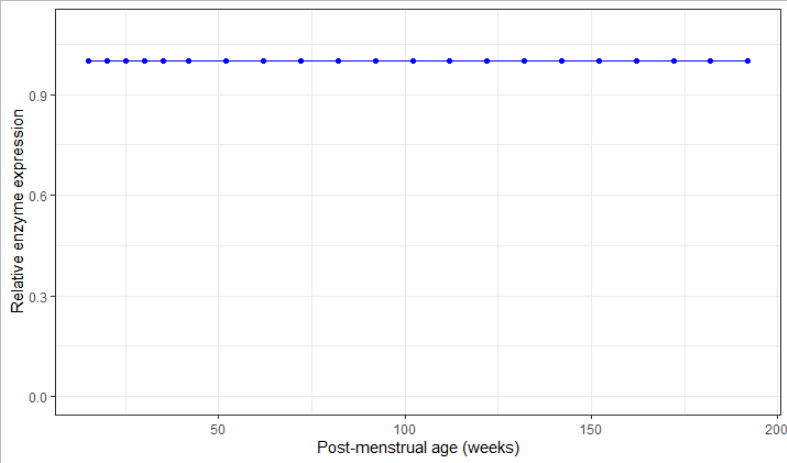

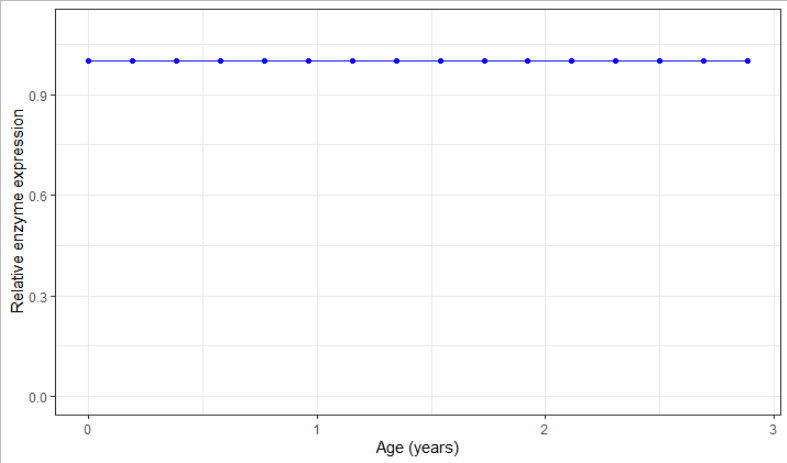


**CYP2C8 expression with age in PK-Sim* (imatinib)**
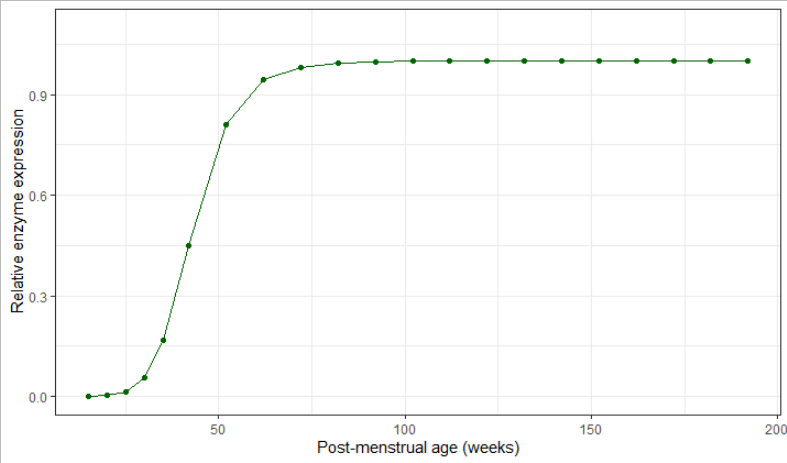

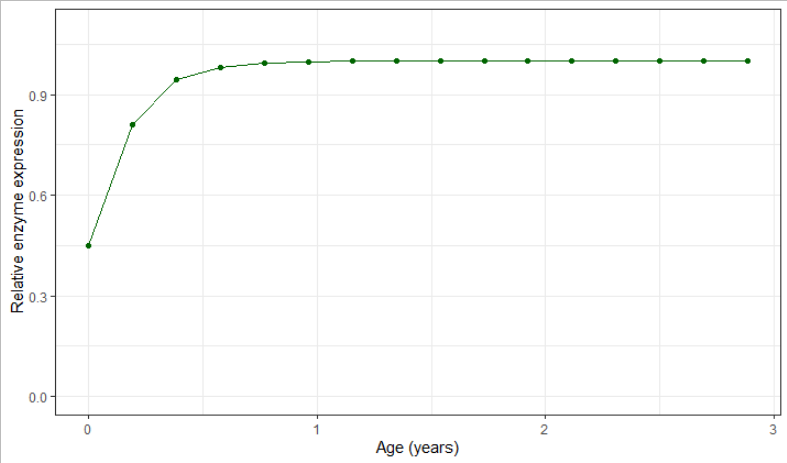


*Enzyme maturation plots were generated using the post-menstrual age maturation functions reported by PK-Sim. More detailed information regarding the maturation functions for each enzymes and the changes in enzyme expression with age can be found at the https://github.com/Open-Systems-Pharmacology/OSPSuite.Documentation page in the ‘*’PK-Sim Ontogeny Database Version 7.3.pdf*” document.

(1) PK-Sim Ontogeny Database Version 7.3.pdf. Accessed 4 April 2024. Available at: https://github.com/Open-Systems-Pharmacology/OSPSuite.Documentation/blob/master/PK-Sim%20Ontogeny%20Database%20Version%207.3.pdf

**Adult PBPK model results**

Imatinib


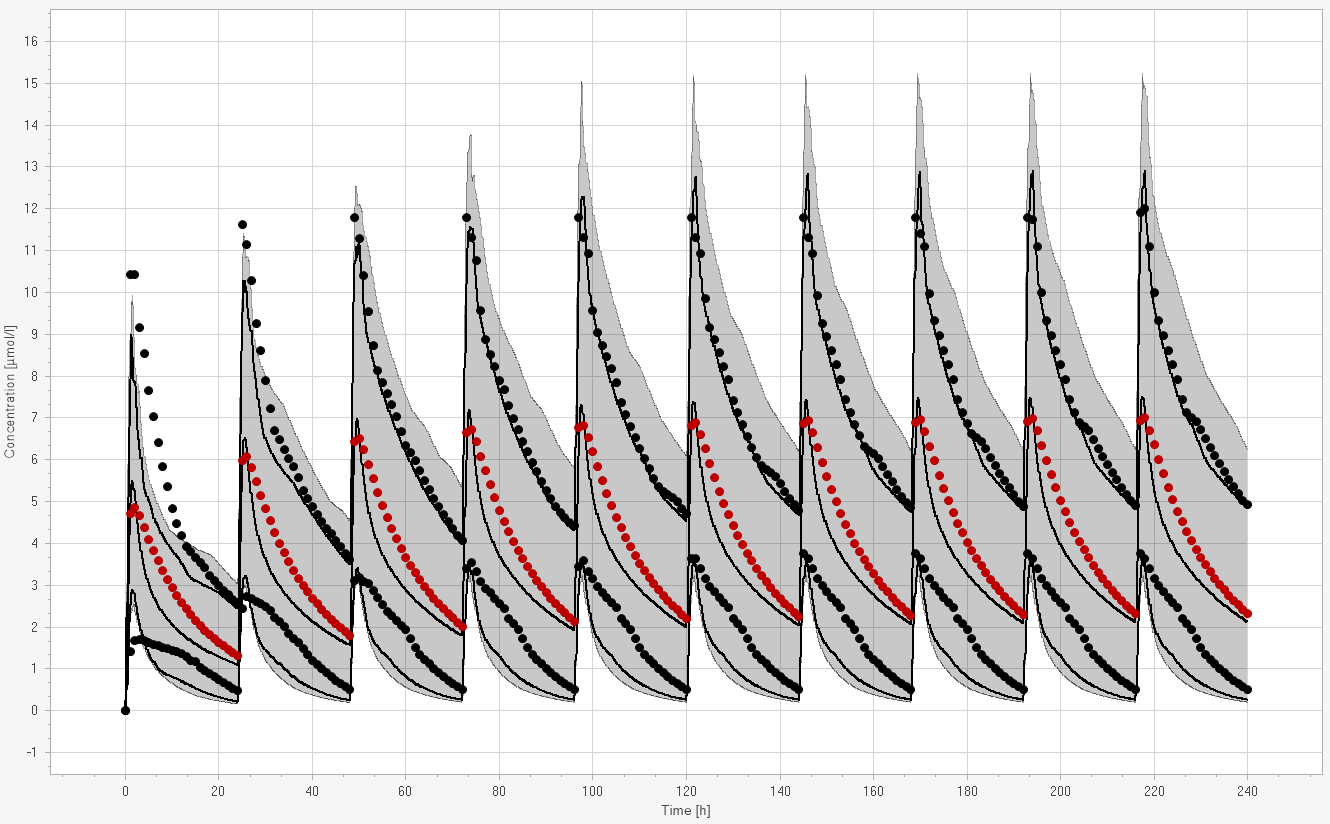


NDMI
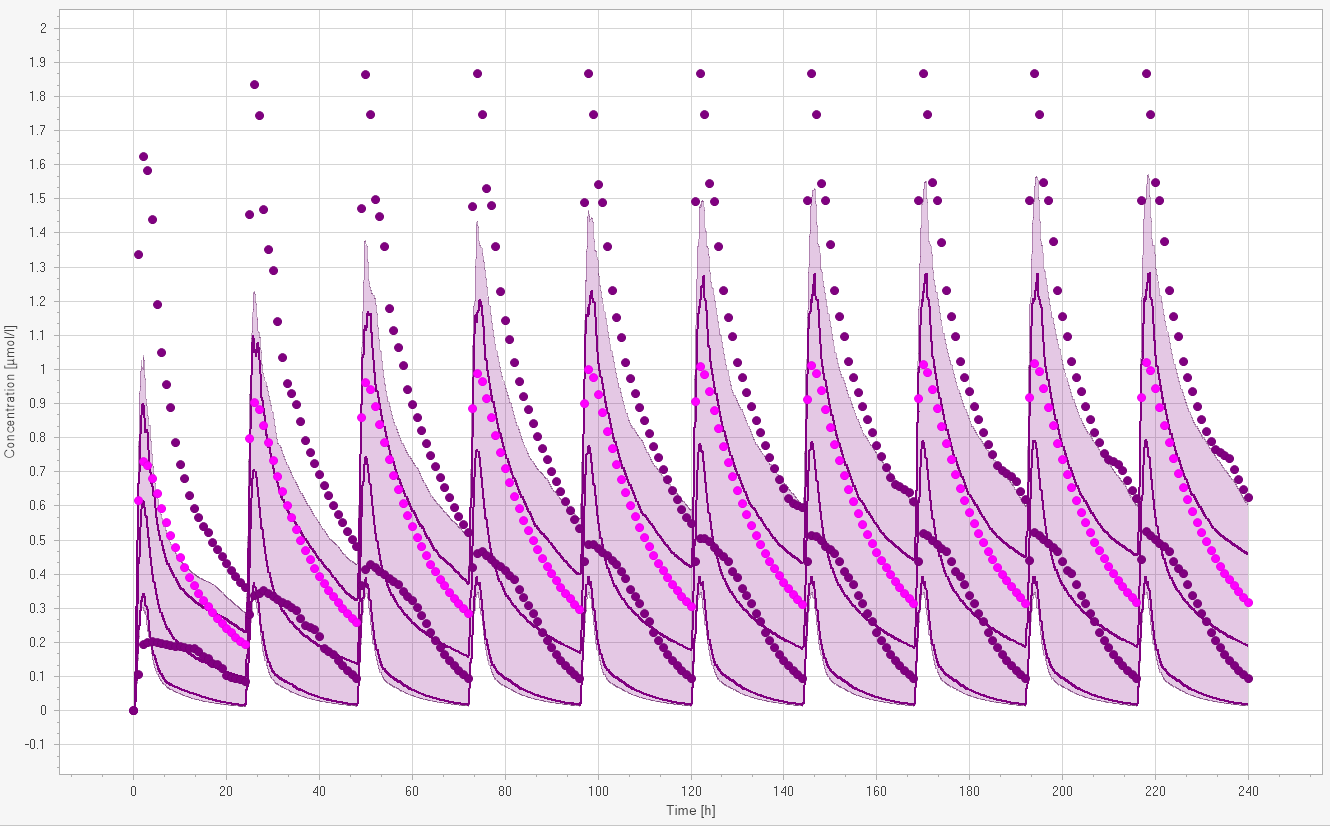


Sunitinib


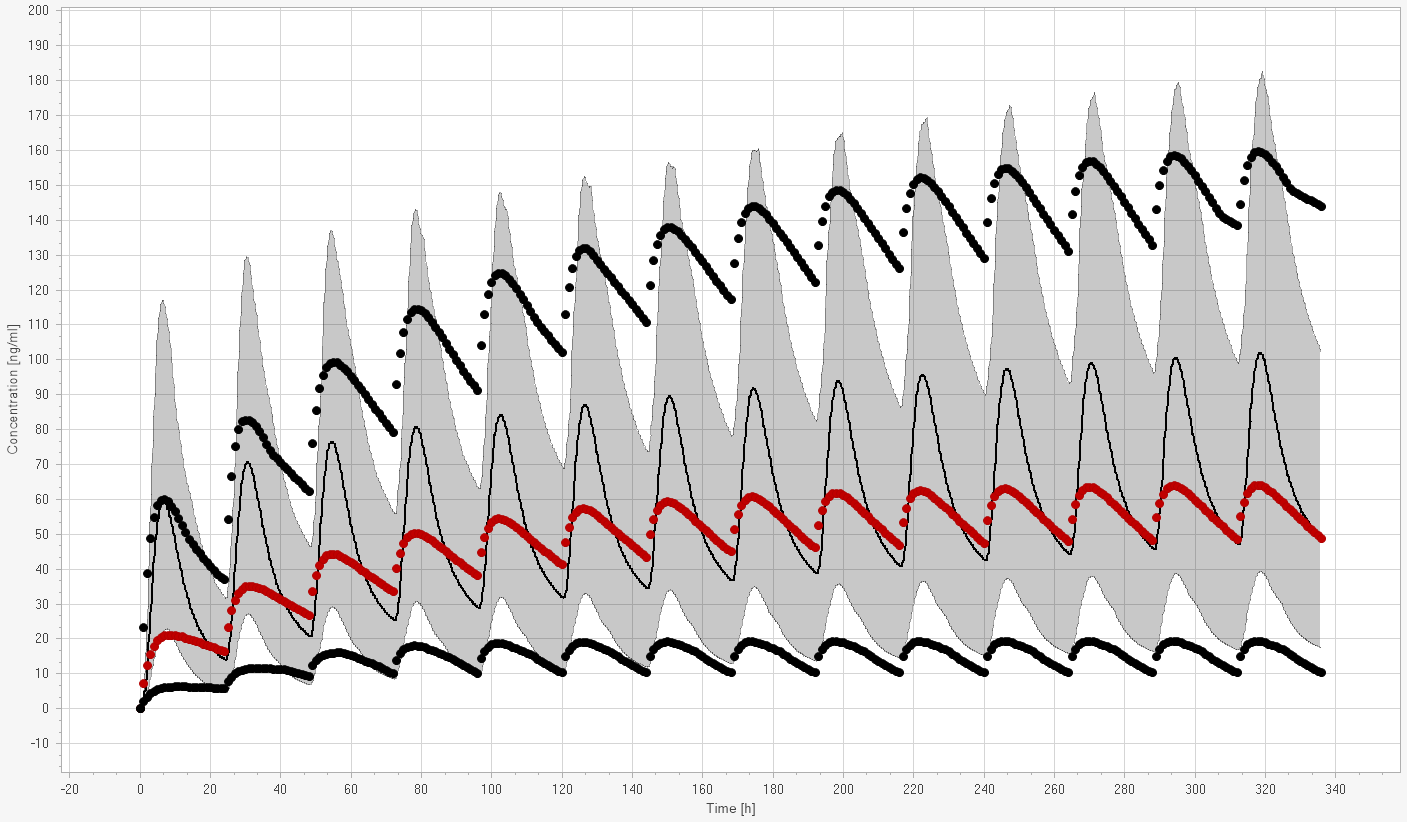


SU12662


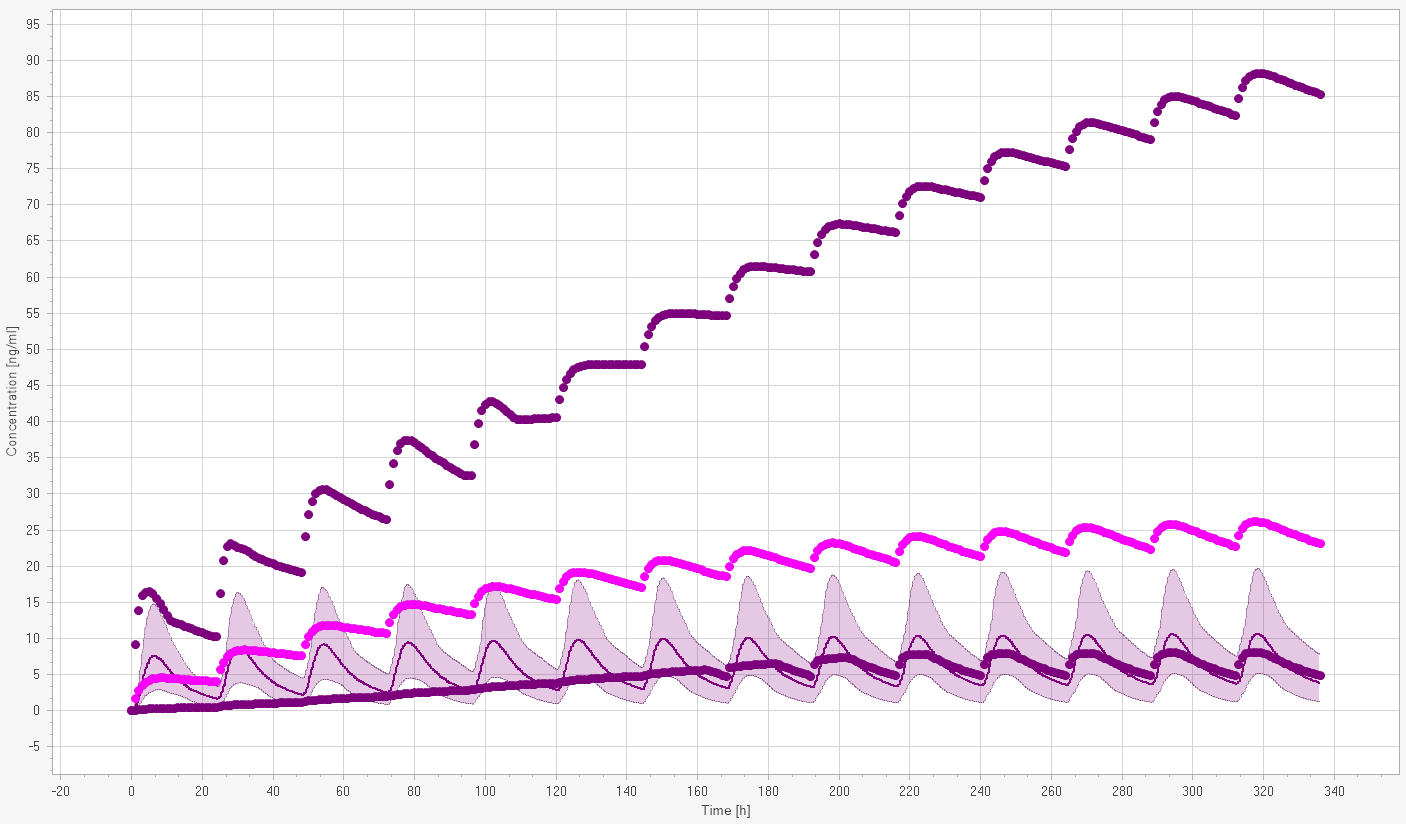


Pazopanib (200 mg)


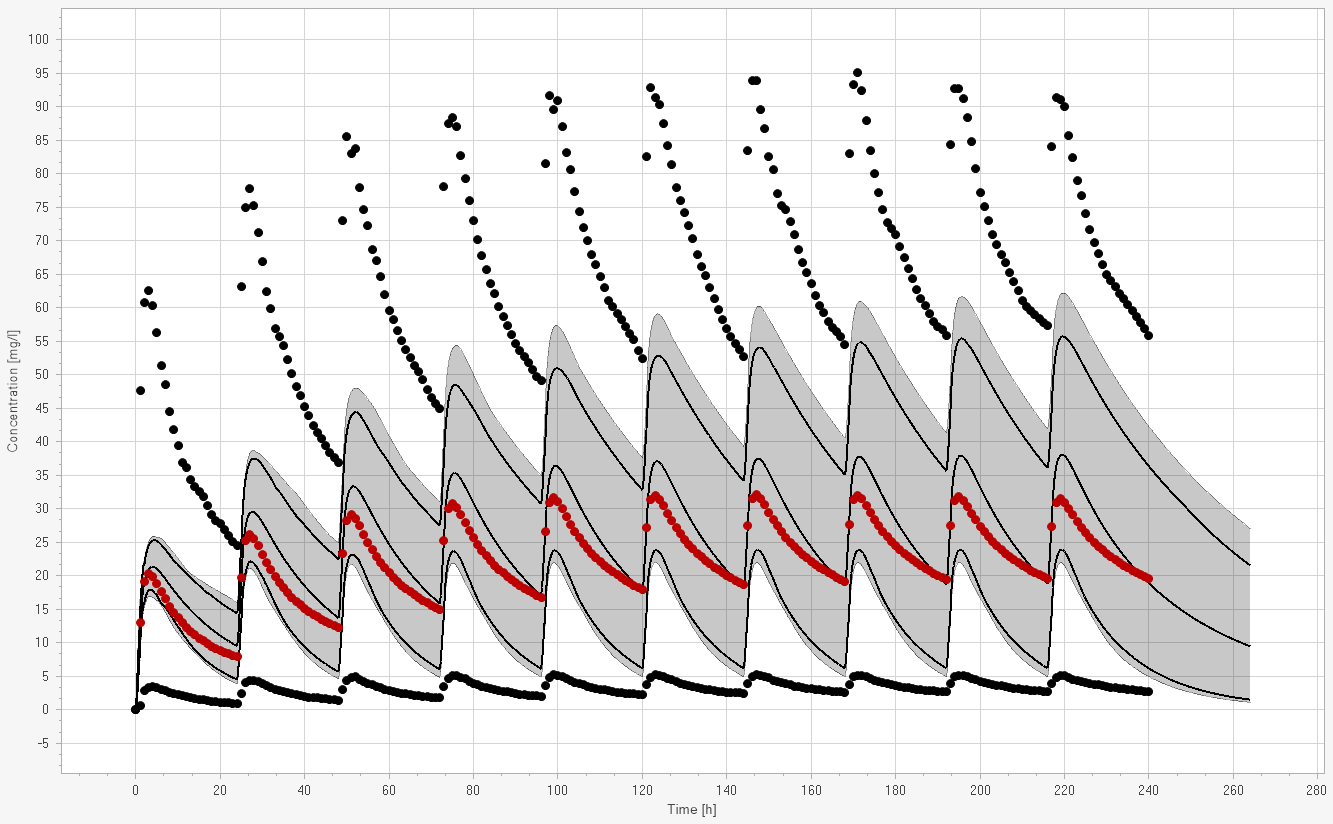


Pazopanib (400 mg)


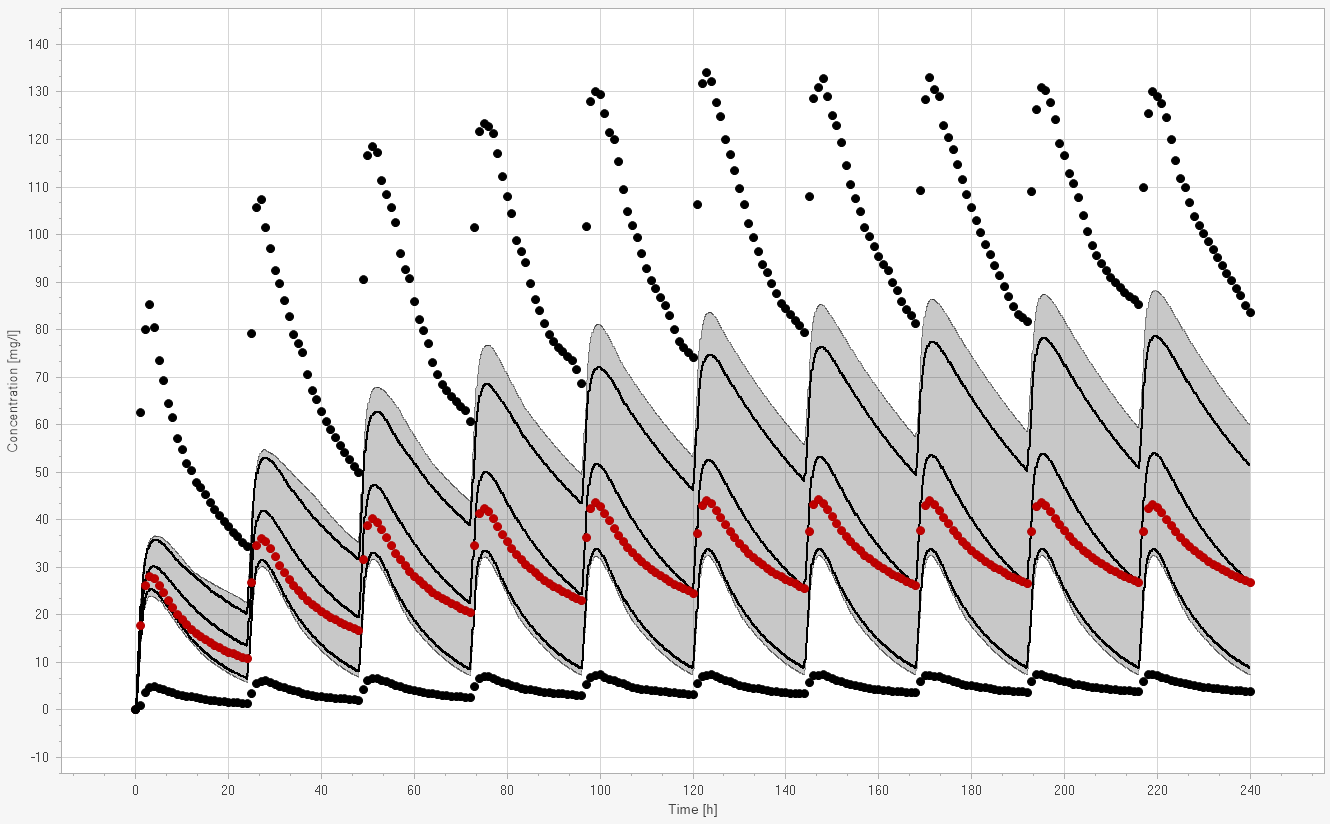


Pazopanib (800 mg)


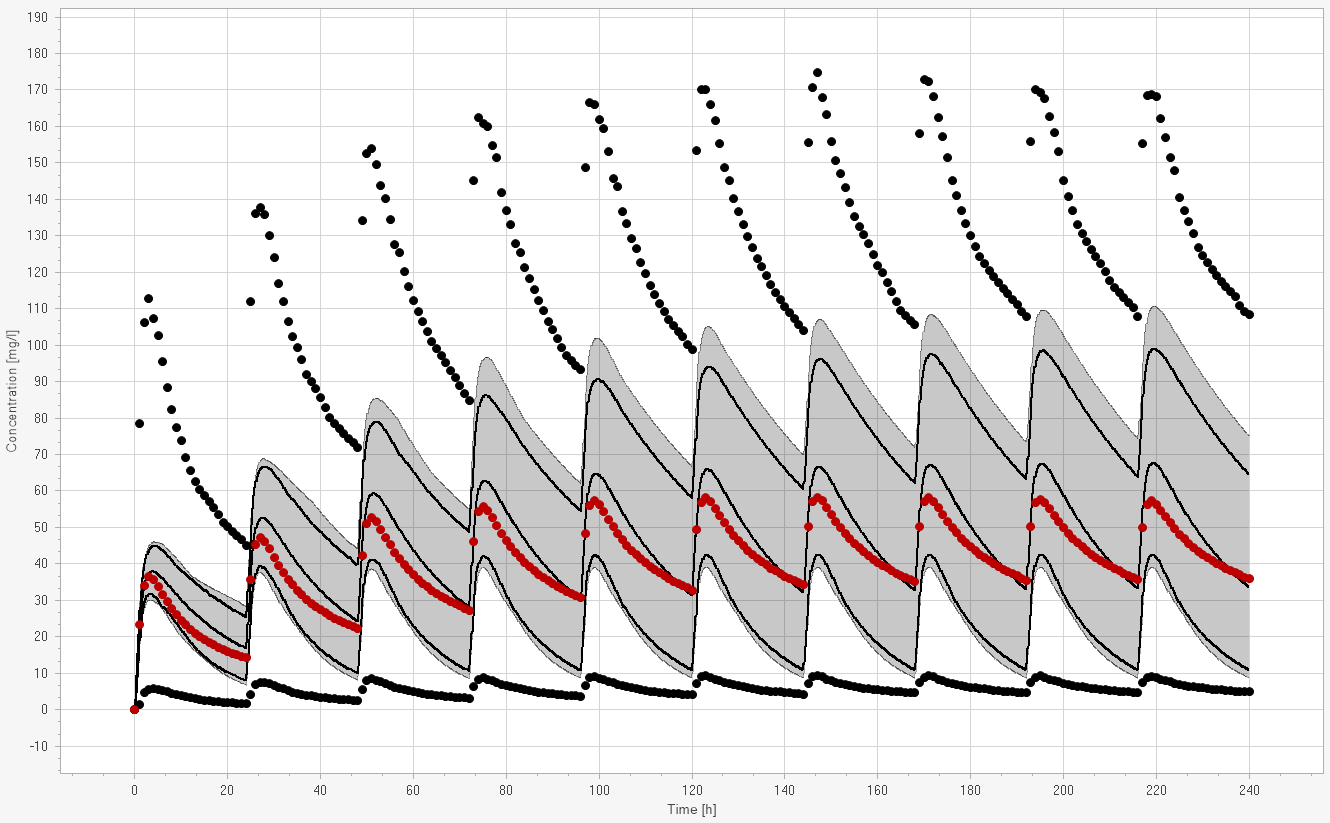


**Online Resource 1: Simulated and observed concentration for the adult PBPK models**. The solid circles (•) represent observed (simulated with adult popPK model) mean, 2.5th and 97.5th percentiles. The solid lines (---) are the simulated mean (with PBPK model), 2.5th and 97.5th percentiles of the observed data. The shaded areas correspond to the simulated (with PBPK model) 95% confidence intervals (n = 100). *PBPK physiologically-based pharmacokinetic mode*.
